# Levamisole-Adulterated Cocaine-Induced Thrombotic Vasculopathy With Negative Serology

**DOI:** 10.7759/cureus.26594

**Published:** 2022-07-05

**Authors:** Shawn Y Sunu, Kartik Dhaduk

**Affiliations:** 1 Family Medicine, Geisinger Health System, Wilkes-Barre, USA; 2 Internal Medicine, Geisinger Health System, Wilkes-Barre, USA

**Keywords:** negative serology, cocaine vasculitis, thrombotic vasculopathy, vasculitis, levamisole

## Abstract

Substance abuse is an important public health issue in the United States. The prevalence of cocaine use is wide, and it is noted to be adulterated with a substance called levamisole, which can increase the bulk and possibly potentiate cocaine’s euphoric effect. Literature shows that levamisole-induced vasculopathy has a strong association with antineutrophil cytoplasmic antibodies (ANCA) antibodies. However, we report a case of biopsy-confirmed levamisole-related thrombotic vasculopathy with negative perinuclear antineutrophil cytoplasmic antibody (p-ANCA) and cytoplasmic antineutrophil cytoplasmic autoantibody (c-ANCA) antibodies. Our case highlights the serious consequences of substance abuse. Here, we provide educational value and encourage physicians to keep the differentials broad when encountering a dermatological case in patients with cocaine use and highlight the importance of skin biopsy for the diagnosis and appropriate management.

## Introduction

Cocaine is a highly potent and addictive illegal stimulant that can be ingested in multiple ways. A recent study from the National Surveys on Drug Use and Health database, which includes self-reported data from adults (age 18 or older) showed the annual average estimated prevalence of cocaine use in adults in the United States to be 2.14% for 2018-2019 [1]. However, illicit cocaine is often not sold in the purest form on the streets. A United States-based study in 2011 revealed that 78% cocaine positive urine samples also contained levamisole [2]. Levamisole, an immunomodulatory and anthelminthic agent, is often found in adulterated cocaine to dilute the purity of cocaine and reportedly potentiates cocaine’s euphoric effects [3,4]. Levamisole was withdrawn from its medical use as an immunomodulatory agent in the United States in 2000 because of adverse effects including agranulocytosis, purpura, skin necrosis, and arthralgia [5].

Levamisole-induced skin lesions are typically seen over the extremities, face, and ear with positive antineutrophil cytoplasmic antibodies (ANCA) serologies, typically high titers of perinuclear antineutrophil cytoplasmic antibody (p-ANCA) rather than cytoplasmic antineutrophil cytoplasmic autoantibody (c-ANCA) antibodies [4]. Here, we present a case of biopsy-confirmed levamisole-related thrombotic vasculopathy with negative p-ANCA and c-ANCA antibodies in a patient with active cocaine use.

## Case presentation

**Figure 1:**
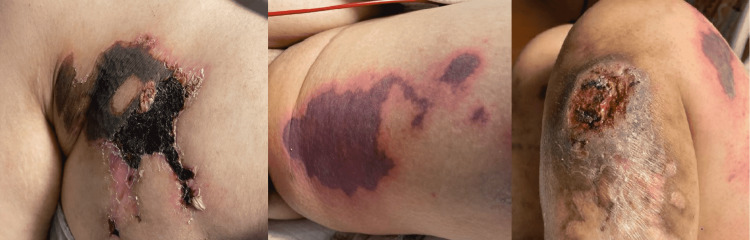
Images showing various stages of necrotic, purpuric skin lesions.

A 40-year-old female with a medical history of methicillin-resistant *Staphylococcus aureus* (MRSA) bacteremia and intravenous (IV) drug use presented to the hospital due to a multitude of non-healing lesions in various stages. The first lesion began five days before presentation over the left anterolateral region of her shin right below her knee after IV drug use (the initial lesion was at a different site from the known injection site). Similar lesions were then later noticed by the patient over her breasts and buttocks (lesions were also not at the known injection site). The patient also reported subjective fevers before admission and lower extremity swelling around the initial lesion. However, the patient denied any trauma, insect bites, or exposure to ticks. The patient also denied a productive cough, shortness of breath, chest pain, diarrhea, dysuria, and urinary discoloration. On admission, the patient was febrile to 101.1^o^ F but hemodynamically stable in no acute distress. Pertinent positive physical exam findings included an approximately 10 cm cratered ulcer located on her left lower extremity and additional purpuric eschar wounds on her right breast and buttocks bilaterally (Figure 1). Pertinent negative physical exam findings included no visual changes, no focal neurological deficits, clear lung sounds, no murmurs, no abdominal tenderness, no hepatosplenomegaly, and no signs of subacute bacterial endocarditis (i.e., Osler nodes, Roth spots).

The differential diagnosis at the time of her admission included cellulitis secondary to needle stick or insect bites, bacterial or fungal infection, cocaine-induced vasculitis or vasculopathy, other autoimmune vasculitides, pyoderma gangrenosum, or malignancy. Initial labs revealed a normal creatinine, a normal glomerular filtration rate (GFR), a normal leukocyte count, a normal platelet count, and a hepatic function panel with a mildly elevated liver alkaline phosphatase level. Due to both her history of MRSA bacteremia and her clinical presentation, additional infectious work-up labs were ordered that included an elevated procalcitonin, a normal lactic acid, and a negative admission respiratory viral pathogen panel. However, as we could not rule out a possible vasculitis as well, an inflammatory workup was also ordered that included a markedly elevated C-reactive protein, a minimally elevated erythrocyte sedimentation rate, a negative c-ANCA, and a negative p-ANCA. As a result of the patient’s clinical picture and laboratory results, the patient was started on piperacillin-tazobactam and vancomycin on presentation. Initial blood cultures grew MRSA with sensitivities to tetracycline and vancomycin. Subsequent repeat blood cultures showed no growth. Additional workup included cocaine-positive urine toxicology and a negative sexually transmitted infections (STI) panel workup (rapid plasmin reagin (RPR) negative, gonorrhea-chlamydia negative, HIV negative, hepatitis C antibody positive, hepatitis C RNA quantitative negative). All laboratory values are noted in Table 1. Later on in her admission, a transesophageal echocardiogram showed a left ventricular ejection fraction of 55% with no abscesses, valvular mass, or vegetations. A skin biopsy of the right thigh was also done for definitive diagnosis, which revealed vessels of various sizes throughout the sampled dermis that showed luminal occlusion by fibrin thrombi without significant inflammation which was suggestive of levamisole-associated thrombotic vasculopathy.

**Table 1:** Laboratory test and values with reference ranges.

Laboratory Test	Laboratory Value	Reference Range
Creatinine	0.8 mg/dl	0.5-1.0 mg/dL
Glomerular Filtration Rate	> 90 mL/min	> 60 mL/min
White Blood Count	7.51 K/uL	4.00-10.80 K/uL
Platelet Count	295 K/uL	140-400 K/uL
Alkaline Phosphatase	147 U/L	35-130 U/L
Procalcitonin	6.01 ng/mL	< 0.10 ng/mL
Lactic Acid	1.5 mmol/L	0.4-2.0 mmol/L
Blood Cultures	Methicillin-resistant *Staphylococcus aureus*	NA
Respiratory Pathogen Panel	Negative	NA
C-Reactive Protein	199 mg/L	< 5 mg/L
Erythrocyte Sedimentation Rate	32 mm/hour	< 20 mm/hour
Centrally Accentuated Cytoplasmic Antibody Test (c-ANCA)	Negative	NA
Perinuclear Antineutrophil Cytoplasmic Antibody Test (p-ANCA)	Negative	NA
Urine Toxicology	Cocaine Metabolite	NA

As the patient improved clinically and her repeat blood cultures were negative, the discharge plan included setting up an appointment with a methadone clinic, a six-week course of doxycycline for her MRSA infection, and a close four-week follow-up with infectious disease.

## Discussion

The mechanism of levamisole-induced vasculitis is not fully understood, but there are still some commonalities that have been seen through recent studies suggesting a role of autoimmunity. A systemic review with 192 cases reported ANCA positivity in 93.8% cases with 43% of cases having both c-ANCA and p-ANCA positivity [6]. Other common associated antibodies reported are antinuclear (ANA), anti-double-stranded DNA antibodies (anti dsDNA), and antiphospholipid (APL) antibodies such as lupus anticoagulant and anti-cardiolipin antibodies [3,4]. Our patient was tested for both c-ANCA and p-ANCA, which were negative. We did not test for ANA, anti-ds-DNA, and APL antibodies.

Biopsies of the skin lesions in levamisole-induced vasculitis typically show occlusive thrombotic vasculopathy without vasculitis, leukocytoclastic vasculitis, or thrombotic vasculitis [3,4,6]. A systemic review study reported the prevalence of vasculitis to be 49% and thrombotic vasculopathy to be near 42% in skin biopsies [6]. Our patient showed luminal occlusion by fibrin thrombi without significant inflammation in histopathological evaluation from a skin biopsy from the thigh.

Directly testing for levamisole is limited considering its short half-life (5.6 hours) and unavailability through routine toxicology screening. Regarding the treatment for levamisole-induced vasculopathy, this should include withdrawal of the inciting drug, supportive care, targeted antibiotics for superimposed bacterial skin infections, and wound debridement if needed. Beyond the discontinuation of the causative agent- levamisole contaminated cocaine, appropriate management of this vasculopathy is yet to be established. Additionally, there is a possible role of systemic corticosteroids and literature suggests its potential benefit in patients with striking signs of inflammation and elevated CRP levels [7-9].

This case highlights the serious consequences of adulterated cocaine use. With rising numbers of cocaine users, clinicians should be aware of this important public health aspect and be able to recognize the characteristic purpuric skin lesions over extremities, face, and ear in such population that may represent an etiology secondary to levamisole adulteration.

## Conclusions

We provide an educational value with this case report and encourage physicians to keep a broad differential diagnosis when encountering a dermatological disease in the presence of cocaine use. Additionally, this case report is unique as our patient did not have ANCA-associated vasculitis, which is otherwise a usual finding of levamisole-induced vasculitis. In such situations, histopathological confirmation of diagnosis would be helpful to provide appropriate supportive care to prevent unnecessary resource utilization. 
